# Long-term exercise training improves memory in middle-aged men and modulates peripheral levels of BDNF and Cathepsin B

**DOI:** 10.1038/s41598-019-40040-8

**Published:** 2019-03-04

**Authors:** Adrián De la Rosa, Elisabeth Solana, Rubén Corpas, David Bartrés-Faz, Mercè Pallàs, Jose Vina, Coral Sanfeliu, Mari Carmen Gomez-Cabrera

**Affiliations:** 1 Freshage Research Group, Department of Physiology, Faculty of Medicine, University of Valencia and CIBERFES, Fundación Investigación Hospital Clínico Universitario/INCLIVA, Valencia, Spain; 2 Departament de Medicina, Facultat de Medicina, Universitat de Barcelona, and Institut d’Investigacions Biomèdiques August Pi i Sunyer (IDIBAPS), Barcelona, Spain; 3 Institut d’Investigacions Biomèdiques de Barcelona (IIBB), CSIC, IDIBAPS and CIBERESP, Barcelona, Spain; 4 Unitat de Farmacologia i Farmacognòsia, Facultat de Farmàcia i Ciències de l’Alimentació, Institut de Neurociència, Universitat de Barcelona, Barcelona, Spain

## Abstract

Aging is accompanied by a decline in memory and other brain functions. Physical exercise may mitigate this decline through the modulation of factors participating in the crosstalk between skeletal muscle and the brain, such as neurotrophins and oxidative stress parameters. We aimed to determine whether long term exercise training (35 ± 15 years) promotes memory maintenance in middle-aged men, and to characterize the changes in neurotrophic factors and lipid oxidation markers in peripheral blood samples in both middle-aged and young men. The neuropsychological analysis showed significant improvements in memory through the Free and Cued Immediate Recall tests, in the middle-aged trained individuals when compared to the sedentary ones. We found a significant decrease in the resting serum BDNF and plasma Cathepsin B (CTSB) levels in the trained groups at both middle and young ages. BDNF and CTSB levels were inversely correlated with weekly hours of exercise. We also found a significant decrease in plasma malondialdehyde, an index of lipid peroxidation, in middle-aged and young trained subjects. The positive impact of long-term exercise training by delaying the onset of physiological memory loss and the associated neurotrophic and redox peripheral modulation, suggests the effectiveness of exercise as preventive strategy against age-related memory loss and neurodegeneration.

## Introduction

Physical exercise is closely related to the cognitive function through a cascade of cellular and molecular processes that promote angiogenesis, neurogenesis and synaptogenesis thus enhancing learning, memory, and brain plasticity^1^.

Among the peripheral factors considered modulators of the aforementioned mechanisms, are Cathepsin B (CTSB) and Brain-Derived Neurotrophic Factor (BDNF). BDNF can be synthesized in peripheral tissues such as skeletal muscle, liver, adipose tissue, endothelial and immune cells. However, the brain contributes to 75% of its synthesis under normal conditions^2,3^.

BDNF is a promoter of several aspects of brain development which are known to be mediated by its tyrosine kinase receptor B (TrkB) in the hippocampus and cerebral cortex^4,5^, and carried out through complex signaling pathways^6^. An increase in BDNF concentrations is associated with an increase in hippocampal size and an improvement in the performance of spatial memory and learning^7^. BDNF and TrkB expression in the hippocampus and temporal cortex decrease over the years, in humans, increasing the risk of suffering from different neurodegenerative pathologies^8^. BDNF levels are increased two to three-fold after acute exercise when compared to resting conditions and correlate positively with improvements in cognitive functions in humans^9–11^. Levels of BDNF have been less characterized after chronic exercise. It has been reported no significant changes^12^ or an increase in resting BDNF levels^13,14^ after exercise training ranging from several weeks to one year. However, long term habitual exercise is associated with lower resting peripheral BDNF levels and better intermediate memory both in middle-aged^15^ and young individuals^16^.

CTSB belongs to the papain superfamily and is the most abundant cysteine protease expressed in all human tissues^17^. CTSB is considered key in neuroprotective lysosomal activation, neuronal survival and, although controversial^18^, it has a significant anti-amyloidogenic activity^19,20^. CTSB is considered as a myokine capable of crossing the blood-brain barrier to mediate processes related to cognition through the induction of doublecortin and BDNF^21^. Exercise training induces CTSB in gastrocnemius, hippocampus, and plasma of mice, monkeys and humans^21^. Interestingly a four weeks exercise-induced increase in CTSB correlates, in healthy young adults, with fitness and hippocampus-dependent memory function^21^.

During aging a decline in brain tissue is accompanied with a decrease in learning, memory and hippocampal neurogenesis^22,23^. Exercise can mitigate these age-related losses. At the end of the 70’s it was found that older adults who regularly engaged in physical activity had a greater psychomotor speed when compared to their sedentary counterpart in tests of simple reaction time and choice^24^. The hippocampus is a determinant in the processes of learning and memory. Erickson and co-workers reported a significant increase in the serum levels of BDNF and in the anterior, left, and right hippocampus size of elderly people who exercised regularly for one year, when compared to the sedentary group^13^. Two years later, it was found that exercise-induced increase in temporal lobe functional connectivity was associated with changes in growth factors in old individuals^25^.

Usually, the studies report positive effects of exercise training in memory tasks and cognitive functions, in older adults with mild cognitive impairment^26,27^, glucose intolerance^28^, and Alzheimer’s disease^29^. However, little is known about the effect of exercise on the onset of physiological memory loss, not during a disease condition, but on the trajectory of normal brain ageing.

Thus, the main purpose of this study was to evaluate the impact of long-term exercise training on memory and peripheral markers related to cognitive function and oxidative stress in healthy middle-aged individuals.

## Methods

### Subjects

Eighty-six healthy men between 17 and 68 years volunteered in this study. The subjects were assigned to four different groups: YSG (Young Sedentary Group; n = 21, age in years: 17–25), YTG (Young Trained Group; n = 16, age in years: 18–25), MSG (Middle-Aged Sedentary Group; n = 25, age in years: 47–67) and MTG (Middle-Aged Trained Group; n = 24, age in years; 46–68). The years of education, smoking habits, and hyperglycemia were considered at recruitment to control for bias between sedentary and trained groups. A summary of the subject’s characteristics is given in Table 1.

**Table 1:** General characteristics of the participants.

	YSG (n = 21)	YTG (n = 16)	MSG (n = 25)	MTG (n = 24)
Age (y)	20.9 ± 2.2	19.9 ± 2.0	56.0 ± 5.9	54.3 ± 6.6
Weight (Kg)	75.7 ± 16.3	70.1 ± 8.9	88.3 ± 13.9	88.8 ± 11.6
Height (m)	1.77 ± 0.10	1.76 ± 0.04	1.76 ± 0.10	1.75 ± 0.10
BMI (kg·m^−2^)	24.2 ± 4.6	22.6 ± 3.2	28.4 ± 3.9	28.9 ± 2.7
Weekly hours of exercise	0.2 ± 0.6	9.3 ± 3.8***	0.6 ± 0.8	5.1 ± 2.6***
Schooling (y)	—	—	14.4 ± 3.3	14.9 ± 3.4
**Conditions, No/total (%)**				
Smoking				
Never	13 (61.9%)	16 (100%)	9 (36.0%)	12 (50.0%)
Former	1 (4.8%)	—	8 (32.0%)	8 (33.3%)
Current	7 (33.3%)	—	8 (32.0%)	4 (16.7%)
Hyperglycemia				
Yes/No	0/21 (0%)	0/16 (0%)	5/20 (20.0%)	3/21 (12.5%)
Medication	0/21 (0%)	0/16 (0%)	2/23 (8.0%)	1/23 (4.2%)

Abbreviations: BMI, body mass index.

All values are expressed as the means ± SD. YSG (Young Sedentary Group), YTG (Young Trained Group), MSG (Middle-Aged Sedentary Group) and MTG (Middle-Aged Trained Group). ***p < 0.001 vs sedentary group. Statistical significance was assessed using a one-way ANOVA test.

The young trained participants (YTG) exercised regularly for the last seven years, although the frequency, duration and intensity of their exercise were varied. The sports practiced by the YTG included tennis, running, football and/or taekwondo. All participants in the MTG group were amateur rugby players who have been practicing it during long time. The players reported an average of 35 ± 15 years of practice. Examining veteran athletes, i.e. those who exercised a large part of their lives, may provide novel insight to understand whether exercise training is associated with neuroprotection and the molecular mechanism involved on it. The details of the medical history, life style, training frequency and playing experience were obtained by a neuropsychologist and/or trained nurse. Subjects were excluded if they reported a history of severe disease, pain, cognitive deficiencies, cranioencephalic trauma, or were taking neuroactive or psychoactive drugs or antioxidants.

In the sedentary groups, subjects who reported more than 150 weekly minutes of low intensity physical exercise in the short version of the International Physical Activity Questionnaire (IPAQ), were also excluded.

All subjects were informed verbally and in writing about the nature of the study, including all potential risks. Written informed consent was obtained prior to participation. All procedures were conducted in accordance with the principles of the World Medical Association’s Declaration of Helsinki, and the protocols were approved by the Ethics Committee of the Hospital Clínic, Barcelona, Spain. Written informed consent for all procedures was obtained from all subjects. We recruited only one subject under the age of 18 years in the young group. He was 17 years old. In this case we obtained his informed consent from a parent.

### Sampling

After overnight fasting, venous blood samples were collected between 8:00 and 10:00 a.m. Blood samples were drawn from the antecubital vein in tubes containing EDTA as anticoagulant to obtain plasma or dry tubes to obtain serum. Blood was allowed to coagulate for 60 minutes (10 minutes at room temperature and 50 minutes on ice). Plasma and serum were centrifuged at 1,500 × g, 15 minutes and stored at −80 °C until analyses.

### Pepipheral BDNF

To study BDNF in peripheral blood, serum is generally preferred to plasma and whole blood. This is due to the fact that blood serum has been the conventional standard for most biochemical analysis^3^. Serum BDNF levels were measured using an ELISA kit (BiosensisRapid^TM^ Mature BDNF). Peripheral BDNF concentration shows enormous variability due to several methodological factors which result in variations in BDNF levels in human serum. Two important factors are the time the blood is left to clot prior to serum extraction and the temperature at which clotting occurs. Both of them were strictly controlled in our study.

### Peripheral Cathepsin B

Plasma Cathepsin B levels were measured using an ELISA Kit (Abcam Human Cathepsin B) following the manufacturer’s instructions.

### Plasma lipid peroxidation

Plasma lipid peroxidation was determined following a method previously described^30^. This method is based on the hydrolysis of lipoperoxides in plasma and subsequent formation of an adduct between thiobarbituric acid and MDA (thiobarbituric acid–MDA2). This adduct was detected using high-performance liquid chromatography in reverse phase and quantified at 532 nm.

### Plasma protein carbonylation

The procedure to quantify total protein carbonyls using the OxyBlot kit was densitometry of the Oxyblot and Ponceau staining followed by finding the ratio between the total density in the Oxyblot and the Ponceau.

### Neuropsychological assessment

We performed neuropsychological analysis in all the middle-aged volunteers. All participants showed values into a normal range in a battery of tests covering the main cognitive domains. Attention and psychomotor speed were analyzed with the Trail Making Test Part A and Symbol Digit Modality Test^31^ and Wechsler Adult Intelligence Scale IV Digit Span Subtest^32^. Executive functions were analyzed with the Trail Making Test Part B and Stroop Interference Test^31^. Premorbid intelligence level was analysed with the Wechsler Adult Intelligence Scale IV Vocabulary subtest^32^. Computerized tests from Cambridge Neuropsychological Test Automated Battery (CANTAB software, Cambridge Cognition, UK) were used for spatial working memory, rapid visual information processing and paired associate learning. No statistical differences between MTG and MSG were detected for these tests (data not shown). Furthermore, we evaluated verbal learning and episodic memory by using the Free and Cued Selective Reminding Test (FCSRT)^33^, where we did find differences in the responses between MTG and MSG groups, as shown in the results. FCSRT uses category cues at both acquisition and retrieval in an attempt to enhance recall and ensure semantic encoding. It presents its 16 items as words on stimulus cards (four cards with four items on each card)^34^. Participants were tested individually and told before presentation of test items that they should remember the items so that they could recall them later. Each participant was presented with four items, of different semantic categories, to be recalled. The individual was asked to read all items aloud and then asked to identify the name of each item (e.g., “apple”) when the tester said its category cue (e.g., “fruit”). This procedure continued until all 16 items had been correctly read and identified. After a non-semantic interference task lasting 20 s, the subject attempted to freely recall as many items as possible, in any order. The time allowed for this task was 90 s. The task was stopped if there was no response in 15 s. Items that were not spontaneously remembered were then cued by the examiner (e.g., “Which one was a fruit?”). The category cues were presented to elicit cued recall of only those items that were not retrieved by free recall. This procedure was repeated three times. A 30-min delayed-recall trial was also administered. Each trial was scored for the number of freely recalled items, the number of items recalled after cuing, and the delayed recall (free and cued). Thus, in our study four derived scores were considered: (a) Total immediate free recall (Trial 1 free recall + Trial 2 free recall + Trial 3 free recall; maximum score, 48); (b) Total immediate cued recall (Trial 1 cued recall + Trial 2 cued recall + Trial 3 cued recall; maximum score, 48); (c) Delayed free recall (maximum score, 16); (d) Delayed cued recall (maximum score, 16).

### Statistical analysis

All statistical analyses were performed using SPSS software version 21. The alpha level was set to 0.05, and data results are shown as means ± SDs. Normality of distribution was checked with the Shapiro-Wilk test, and homogeneity of variance was tested by Levene’s statistics. The effect of regular practice of physical exercise and age on BDNF, Cathepsin B, MDA, and protein carbonylation, was assayed using Two-way ANOVA; Bonferroni post-hoc test was used to analyze differences between the group means when there was interaction of both factors, exercise and age. Likewise, when we compared 2 groups, we used a 2-tailed Student’s t test.

We calculated a Pearson’s correlation when data followed a normal distribution. Otherwise, we used a Spearman correlation. Partial correlations were performed for controlling for the effect of additional variables.

## Results

### Characteristics of the subjects

The subject characteristics are summarized in Table 1. There were no significant differences in any anthropometric measurement between the trained groups and the sedentary ones. No differences were found between the trained and the sedentary groups in years of education, smoking habits, and hyperglycemia. As expected, the weekly physical activity level was significantly higher in the active groups compared to the sedentary ones (*p* < 0.001).

### Long-term exercise training is associated with higher memory function in middle-aged rugby players

**Figure 1:**
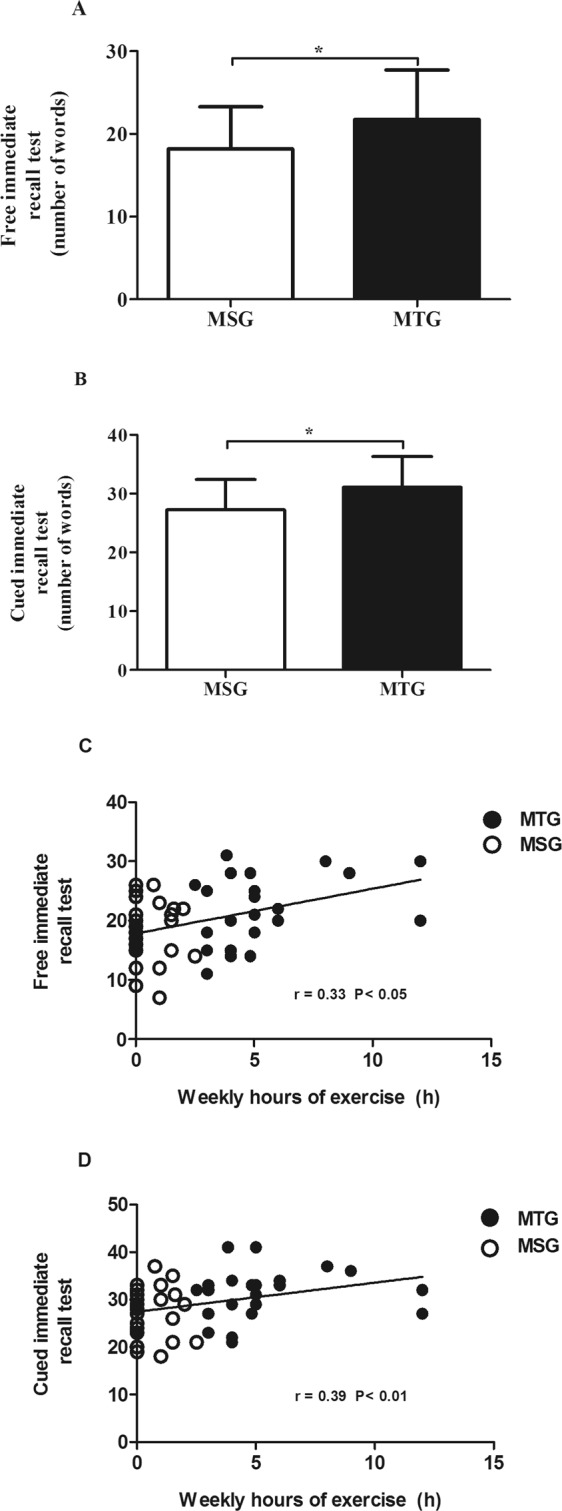
Free and cued selective reminding tests in middle-aged subjects. Effect of a long-term exercise training. FCSRT results in the MSG (Middle-Aged Sedentary Group) and MTG (Middle-Aged Trained Group). Number of words in the total immediate free recall test (**A**) and in the total immediate cued recall test (**B**). Bars represent mean ± SD. Statistical significance was assessed using two-tailed Student’s t-test. *p < 0.05 (**C,D**). Spearman’s correlation test between weekly hours of exercise and number of words in the total immediate free recall test (**C**) and total immediate cued recall test (**D**). For both (**C,D**), values inside the graph indicate the P value of the correlation.

The FCSRT measures verbal learning and memory^35^. This task is particularly sensitive to pathological states especially in early stages of Alzheimer’s disease^36^. It has been reported that free recall impairment on the FCSRT predicts the development of dementia by as much as 5 years in advance of the diagnosis^37^. Our sample of middle-aged rugby players and matched controls were composed of cognitively normal people. However, statistical analysis showed differences between groups. As shown in Fig. 1, the MTG obtained a significant better performance than the MSG in both the free immediate recall (*t*_(47)_ = 2.283, *p* = 0.0270) (Fig. 1A) and the cued immediate recall (*t*_(47)_ = 2.605, *p* = 0.0123) (Fig. 1B). We also determined the free delayed recall (*t*_(47)_ = 1.406, *p* = 0.166) and cued delayed recall (*t*_(47)_ = 1.534, *p* = 0.131) but no significant changes were found between the middle-aged groups.

We also found a positive correlation between weekly hours of exercise and outcomes in the free immediate recall test (r_(49)_ = 0.33, *P* = 0.022) (Fig. 1C) and cued immediate recall test (r_(49)_ = 0.38, *P* = 0.0058; Fig. 1D) in middle aged subjects. These positive correlations were maintained when adjusted for years of education. All the subjects from the middle-aged groups were included in the correlation study.

### Long-term exercise training lowers plasma lipid peroxidation in young and middle-aged trained groups

**Figure 2:**
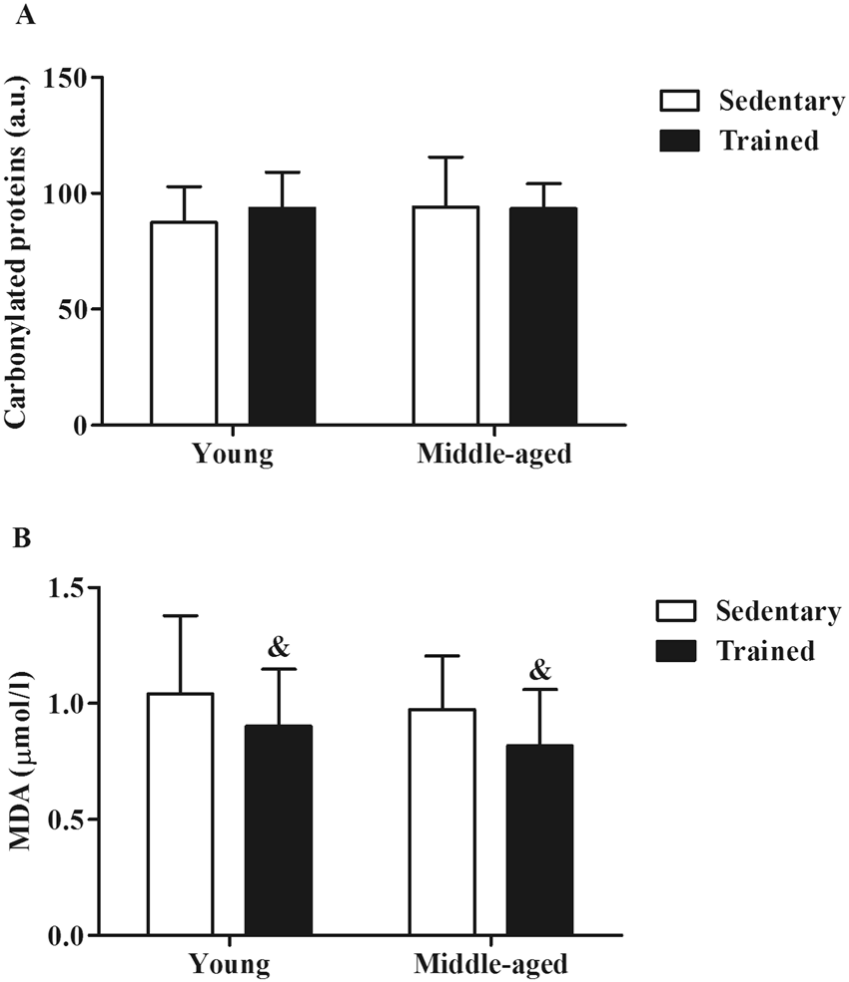
Oxidative damage in young and middle-aged subjects. Effect of a long-term exercise training. Oxidative damage in the YSG (Young Sedentary Group), YTG (Young Trained Group), MSG (Middle-Aged Sedentary Group) and MTG (Middle-Aged Trained Group). (**A**) Densitometric analysis (a.u.: arbitrary units) for plasma protein carbonyl levels measured by Western Blotting. (**B**) Plasma MDA levels measured by HPLC. Bars represent mean ± SD. Statistical significance was assessed using Two-way ANOVA. ^&^p < 0.05 factor exercise training.

It has been previously shown that exercise training induces the antioxidant defense not only in the skeletal muscle but also in blood^30^ and even in brain^2^, which endows trained individuals with a protection against oxidative damage. As shown in Fig. 2A, exercise training does not modify the plasma protein carbonyls in the young or in the middle-age groups. Moreover, we did not find any effect of the age on this parameter. However, the Two-way ANOVA analysis showed an effect of long term exercise training in young and middle-aged individuals in their MDA levels (*F*_(1,77)_ = 6.077; *p* = 0.0159). Figure 2B shows a significant decrease in plasma MDA levels in the trained groups when they were compared with the sedentary ones (ANOVA plots are shown in Supplementary Fig. 1).

### Resting serum levels of BDNF are modulated both by a prolonged period of exercise training and by age in humans

**Figure 3:**
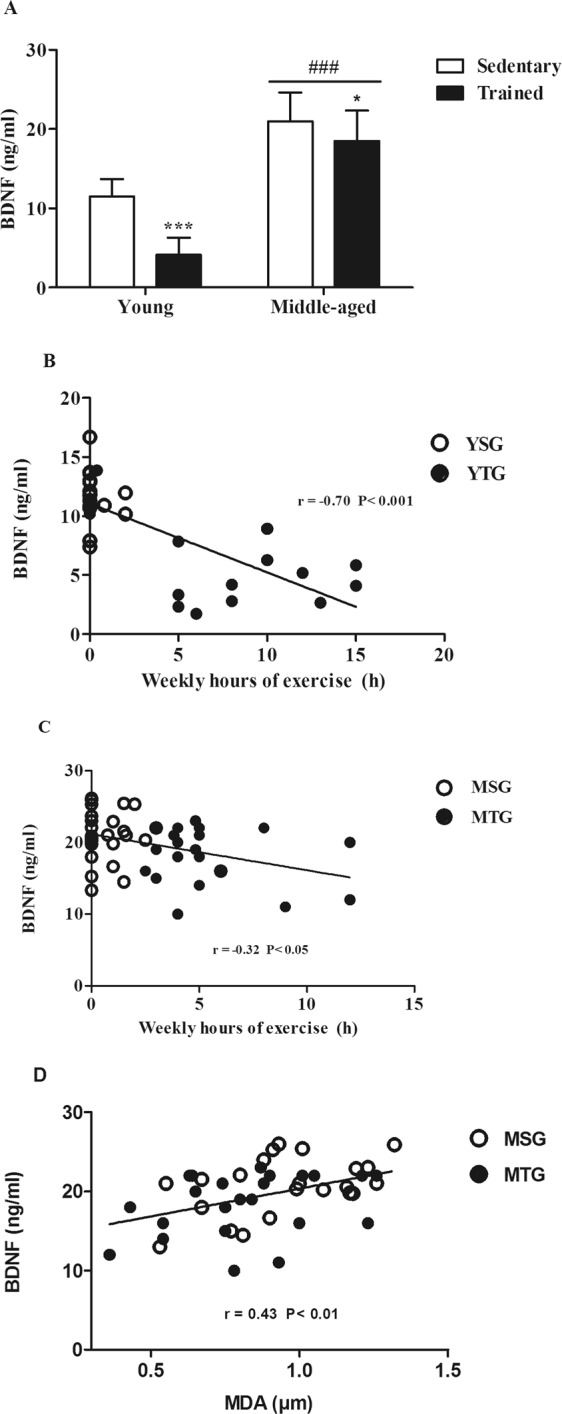
BDNF serum levels and its correlation with malondialdehyde and weekly hours of exercise in young and middle-aged subjects. (**A**) BDNF resting serum levels were determined by ELISA in the YSG (Young Sedentary Group), YTG (Young Trained Group,), MSG (Middle-Aged Sedentary Group) and MTG (Middle-Aged Trained Group). Bars represent mean ± SD. Statistical significance was assessed using the Two-way ANOVA test. Bonferroni post-hoc test: *p < 0.05,***p < 0.001 compared to respective sedentary group; ^###^p < 0.001 compared to respective young group. (**B**,**C**) Spearman’s correlation test between weekly hours of exercise and BDNF resting serum levels in young (**B**) and middle-aged (**C**) individuals. (**D**) Spearman’s correlation between resting serum levels of BDNF and plasma levels of MDA. For B, C, and D, values inside the graph indicate the P value of the correlation.

Comparison of the BDNF levels between the experimental groups was performed using a Two-way ANOVA (Fig. 3A). The analysis showed an effect of exercise training (*F*_(1,80)_ = 50.11; *p* < 0.0001), age (*F*_(1,80)_ = 289.6; *p* < 0.0001) and the interaction between these factors (*F*_(1,80)_ = 11.94; *p* = 0.0009). ANOVA plot confirmed this interaction, showing a higher effect of exercise in YTG than in MTG (Supplementary Fig. 1). In addition, the post-hoc Bonferroni analyses revealed an increase in the BDNF levels with age and a decrease with physical exercise that was more intense in the young group.

It has been previously suggested that there is an inverse relationship between the resting peripheral BDNF level and habitual physical activity or the cardiorespiratory fitness^16,38^. Figure 3B,C show a significant inverse correlation between serum BDNF and weekly hours of exercise in the group of young individuals (r_(33)_ = −0.709, *p* < 0.0001), and in the middle-aged ones (r_(47)_ = −0.32, *p* = 0.026). We did not find any significant correlation between BDNF and the score in the memory tests (data not shown). Finally, Fig. 3D shows a significant positive correlation between BDNF and MDA (r_(45)_ = 0.434, *p* = 0.0029) in the middle-age groups.

### Cathepsin B resting plasma levels are modulated by a prolonged period of exercise training in young and middle-aged subjects

**Figure 4:**
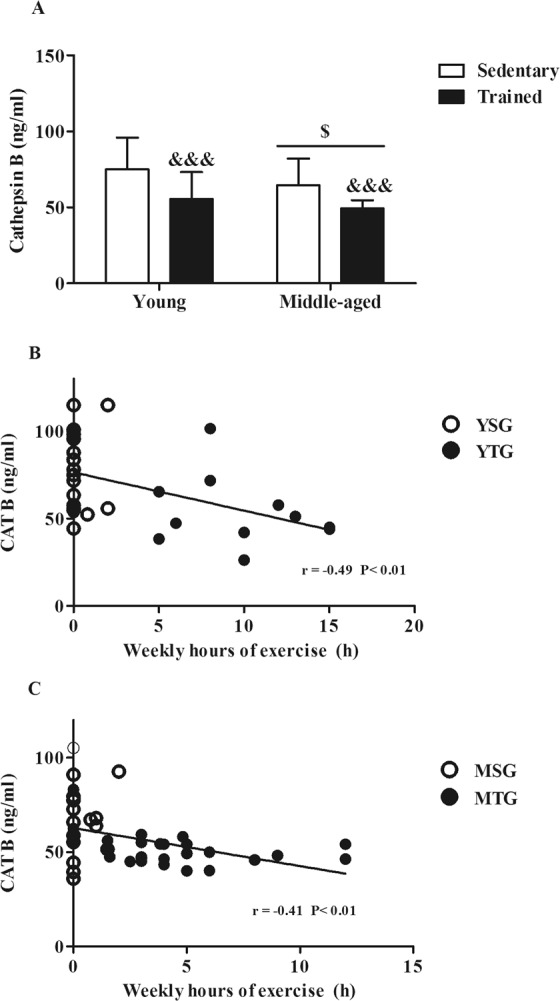
Cathepsin plasma levels and its correlation with malondialdehyde and weekly hours of exercise in young and middle-aged subjects. (**A**) Cathepsin B resting plasma levels were determined by ELISA in the YSG (Young Sedentary Group), YTG (Young Trained Group), MSG (Middle-Aged Sedentary Group) and MTG (Middle-Aged Trained Group). Bars represent mean ± SD. Statistical significance was assessed using Two-way ANOVA. ^$^p < 0.05 factor age; ^&&&^p < 0.001, factor exercise training. (**B**,**C**) Spearman’s correlation test between weekly hours of exercise and Cathepsin B resting plasma levels in young (**B**) and middle-aged (**C**) individuals. For (**B** and **C**), values inside the graph indicate the P value of the correlation.

The lysosomal cysteine protease CTSB is a myokine that is increased in plasma after exercise training in mice, Rhesus monkeys, and humans^21^. It is considered important for the cognitive and neurogenic benefits of running because it enhances the expression of BDNF^21^. We measured the resting plasma levels of CTSB in all the experimental groups. Two-way ANOVA showed significant effects of exercise training (*F*_(1,76)_ = 22.04; *p* < 0.0001) and age (*F*_(1,76)_ = 5.045; *p* = 0.0276) (Fig. 4A and Supplementary Fig. 1). Figure 4B,C show a significant inverse correlation between plasma CSTB and weekly hours of exercise in the group of young individuals (r_(32)_ = −0.49; *p* = 0.004), and in the middle-aged ones (r_(44)_ = −0.41;*p* = 0.005). We did not find any significant correlation between CTSB and the score in the memory tests (data not shown). Thus, CSTB, in response to long term exercise, behaved as BDNF in our study.

## Discussion

Cognitive impairment and dementia have become serious social, economic, and human burdens^39^. Prevention is a key element to counteract the dementia epidemic^40^. A third of Alzheimer’s disease cases worldwide are estimated to be attributable to seven modifiable factors: midlife hypertension and obesity, low education, diabetes, physical inactivity, smoking, and depression^39^. These data provide prevention opportunities. However, after many negative dementia trials, the focus of the preventive strategies has shifted to pre-symptomatic and pre-dementia disease stages and at-risk states when intervention might not be too late.

The major aim of our study was to detect whether long term exercise training in a team sport, may improve cognitive responses at an age when subtle age-related loss could be detected. We speculate that the detected memory improvement is associated with a delay in the onset of physiological memory loss. Therefore, the study of therapies and mechanisms promoting normal memory maintenance might help to design specific programs to improve memory in disease status, such as Alzheimer’s. This is why we selected middle-aged individuals as our target study population. Among the middle-aged individuals, we included a very valuable group, rugby players that reported an average of 35 ± 15 years of practice.

Traditionally, most of the studies showing that the cognitive function is improved with exercise have been performed after an acute bout of exercise^10,41^ and/or chronic aerobic exercise^13,42^. However, no evidences for the benefits of a long-term practice of team sports are available in the literature. The evidence in support of the effects of aerobic exercise to improve memory in humans has not been established convincingly. The vast majority of studies assessing the effects of cardiovascular exercise on cognition, have primarily employed neuropsychological tasks targeting mainly attention, decision-making and speed processing (e.g. simple or choice reaction time). In contrast, less emphasis has been placed on investigating the effects of this type of exercise on cognitive tasks involving, for instance, memory^13^.

The FCSRT measures verbal learning and memory. It emphasizes encoding specificity during learning and recall^35^. FCSRT is used widely to identify very mild dementia^43^ because free recall impairment predicts the development of dementia by as much as 5 years in advance of the diagnosis^37^. There is, in general, a decline in most SRT measures with advancing age^44,45^. Our results showing better performance in the free and cued immediate recall tests in the middle-aged trained individuals when compared to the sedentary ones, indicates the positive impact of long-term exercise training by delaying the onset of physiological memory loss on the trajectory of normal brain ageing.

Demographic effects such as education, have been frequently associated with the FCSRT scores^46^. In our study, there were no differences in education, smoking habits or other conditions such as hyperglycemia between the groups.

We then determined the peripheral levels of two well-known mediators of the beneficial effect of exercise on cognition, BDNF and CTSB. BDNF is a neurotrophin that has been identified as a crucial mediator of the benefits of exercise for brain health^47^. It has been reported that the BDNF levels peak in the thirties (30–39 years) and that they tend to decrease slightly at later ages: forties (40–49 years) and fifties (50–59 years)^48^. We have found a significant increase in the resting serum BNDF levels in the middle-aged subjects when compared to the young ones both in the sedentary and in the trained groups. The BDNF resting serum values found in the middle-aged individuals doubled or even increased their levels fourfold when compared with the young ones. So, the age effect was very significant in our study. However, we did not find the same results when analyzing the CTSB peripheral levels. CTSB is considered as a myokine capable of crossing the blood-brain barrier to mediate processes related to cognition through the induction of BDNF^21^. We found moderately lower levels of CTSB in the middle-aged groups and exercise training similarly decreased CTSB levels at both ages. As far as we know, there are not studies in which changes in CTSB levels have been studied during aging. Our results show that regarding age, the peripheral levels of CTSB and BDNF do not follow the same pattern.

Short episodes of high intensity aerobic exercise results in a transient increase in serum levels of BDNF in humans^49^, which return to baseline levels within minutes (30–50 minutes) following exercise cessation and may continue to fall well below baseline levels at 2 and 3 h post-exercise^50,51^. The effect of chronic aerobic exercise (ranging from several weeks to 1 year) although less studied, tends to show that resting BDNF peripheral levels are also increased to some extent after a period of endurance training^14^. However, the relationship between life-long physical activity habits and resting baseline levels of serum BDNF in humans remains unknown. The majority of the findings reported in the literature suggest an inverse association between resting BDNF and habitual physical activity or cardiorespiratory fitness. It has been previously reported that serum BDNF concentration decreases with increasing aerobic power and level of physical activity^16,38,49,51^. Our results are consistent with these studies. We have found a significant decrease in the resting serum BDNF levels both in the young trained group and in the middle-aged rugby players. Previously, Babaei and co-workers found lower serum levels of BDNF and better results when evaluating cognitive function in physically active middle-aged subjects, with respect to their sedentary counterpart^15^.

In the present study, the blood samples were drawn at least 24 h after the last exercise. Therefore, the significant reduction of serum BDNF in trained men can be considered an adaptation to chronic physical activity as opposed to an acute response to one bout of exercise. This idea is reinforced by the correlation of both factors with weekly hours of exercise. Thus, although the peripheral BDNF levels increase immediately after exercise, as a result of a long-term exercise training, the resting serum levels of the neurotrophin are significantly reduced both in the young and middle-aged groups. It could be explained by a training-induced increase in the neurotrophic factor binding sites to repair damage. Exercise induces mechanical and oxidative stress, which causes injury to both muscles and nerves^52,53^. Indeed, physical exercise is known to act as an hormetic stimulus^54^ and BDNF plays a role in repair processes at the site of traumatic injury^55^. BDNF utilization in some tissues could explain the decrement found in the serum samples of the trained individuals. We can speculate that fine-tuning of BDNF signaling, suggested by its lower circulating levels, may provide better neuroprotection in physically active subjects when compared to sedentary controls. Furthermore, exercise may directly activate neuroprotective and neurotrophic signaling pathways downstream of BDNF and modulate pro-BDNF and BDNF binding to TrK receptors^56^.

Regarding CTSB we found a very similar result, a significant decrease on its plasma levels in the trained groups when compared with the sedentary ones. Thus, our results show that the peripheral levels of CTSB and BDNF follow the same pattern in the MTG and the YTG groups. Similarly to BDNF, we cannot discard a higher efficiency of CTSB signaling in trained individuals. Physical exercise is proved beneficial to the brain, although the mechanisms of action are not fully clarified. Beyond the experimental evidence of the involvement of trophic factors such as BDNF and CTSB, it is not clear the association between their circulating levels and the improvement of cognitive function^57^. Furthermore, we cannot rule out the idea that the long-term exercise training improvements in memory, are related to modifications in vascular and/or metabolic risk factor in addition to an improved functional efficiency of neurotrophic signaling^58^. Future research may clarify this issue.

Finally, we measured two markers of muscle oxidative damage in plasma, protein carbonylation and malondialdehyde. The trained groups showed lower levels of lipid peroxidation when compared with the sedentary ones. Classically, it has been considered that oxidative stress increases with older age and that it is the link between aging and memory loss^59^. However, more recently data from our own laboratory show that oxidative damage does not correlate with age, especially in the geriatric population, but rather with the frailty state. This has led us to postulate the “free radical theory of frailty” that proposes that oxidative damage is associated with frailty, but not with chronological age itself^60^.

One of the functional mechanisms of physical exercise is an increase in the antioxidant response that restores redox homeostasis not only in the skeletal muscle but also in the brain^2^. The induction of antioxidant enzymes with exercise training could explain the reduction in the lipid peroxidation in the trained group. Our results showing the positive impact of long-term exercise training by delaying the onset of physiological memory loss on the trajectory of normal brain aging provide promising prevention opportunities for diseases in which this process is a hallmark, like Alzheimer’s disease.

## Conclusion

Cognitive impairment and dementia have become serious social, economic, and human burdens. Prevention is a key element to counteract the dementia epidemic and the preventive strategies may be implemented in pre-symptomatic disease stages when intervention might not be too late. We have found that long term exercise-training improves memory performance in male middle-aged rugby players and decreases peripheral resting levels of the neurotrophins BDNF and CTSB. Exercise can mitigate the age-related brain losses through the modulation of oxidative stress parameters, CTSB, and BDNF circulating levels, these improving neural mechanisms of redox homeostasis, and BDNF and CTSB signaling. Middle-aged individuals had a similar response to young adults to these adaptative changes to long-term exercise training. Our results support the effectiveness of preventive strategies, such as exercise, promoting memory maintenance while we age. This is especially important for diseases in which memory loss is the hallmark symptom such as Alzheimer’s disease.

### Limitations of the study

Although we accounted for potential initial cohort differences in the parameters assessed, our measurements are cross-sectional and therefore we cannot discard some undetected bias. However, we would like to mention that different longitudinal studies have found a direct relationship between the changes in aerobic fitness generated by physical exercise and the results in different functional and cognitive tests^61–65^. We have not been able to include women in our study. More research is needed to analyze the impact of long-term exercise training on neuroprotection in middle-aged women.

## Supplementary information

Supplementary Figure 1

## References

[1] van Praag H, Christie BR, Sejnowski TJ, Gage FH (1999). Running enhances neurogenesis, learning, and long-term potentiation in mice. Proc Natl Acad Sci USA.

[2] Garcia-Mesa Y (2014). Physical exercise neuroprotects ovariectomized 3xTg-AD mice through BDNF mechanisms. Psychoneuroendocrinology.

[3] Pareja-Galeano H (2015). Methodological considerations to determine the effect of exercise on brain-derived neurotrophic factor levels. Clin Biochem.

[4] Christie BR (2008). Exercising our brains: how physical activity impacts synaptic plasticity in the dentate gyrus. Neuromolecular Med.

[5] Park H, Poo MM (2013). Neurotrophin regulation of neural circuit development and function. Nat Rev Neurosci.

[6] Luikart BW (2008). Neurotrophin-dependent dendritic filopodial motility: a convergence on PI3K signaling. J Neurosci.

[7] Tyler WJ, Alonso M, Bramham CR, Pozzo-Miller LD (2002). From acquisition to consolidation: on the role of brain-derived neurotrophic factor signaling in hippocampal-dependent learning. Learn Mem.

[8] Webster MJ, Herman MM, Kleinman JE, Shannon Weickert C (2006). BDNF and trkB mRNA expression in the hippocampus and temporal cortex during the human lifespan. Gene Expr Patterns.

[9] Rasmussen P (2009). Evidence for a release of brain-derived neurotrophic factor from the brain during exercise. Exp Physiol.

[10] Griffin EW (2011). Aerobic exercise improves hippocampal function and increases BDNF in the serum of young adult males. Physiol Behav.

[11] Schmolesky MT, Webb DL, Hansen RA (2013). The effects of aerobic exercise intensity and duration on levels of brain-derived neurotrophic factor in healthy men. J Sports Sci Med.

[12] Schiffer T, Schulte S, Hollmann W, Bloch W, Struder HK (2009). Effects of strength and endurance training on brain-derived neurotrophic factor and insulin-like growth factor 1 in humans. Horm Metab Res.

[13] Erickson KI (2011). Exercise training increases size of hippocampus and improves memory. Proc Natl Acad Sci USA.

[14] Zoladz JA (2008). Endurance training increases plasma brain-derived neurotrophic factor concentration in young healthy men. J Physiol Pharmacol.

[15] Babaei, P., Damirchi, A., Mehdipoor, M. & Tehrani, B. S. Long term habitual exercise is associated with lower resting level of serum BDNF. *Neurosci Lett* 566 (2014). 10.1016/j.neulet.2014.02.011 24572590

[16] Nofuji Y (2008). Decreased serum brain-derived neurotrophic factor in trained men. Neurosci Lett.

[17] Chapman HA, Riese RJ, Shi GP (1997). Emerging roles for cysteine proteases in human biology. Annu Rev Physiol.

[18] Hook V (2005). Inhibition of cathepsin B reduces beta-amyloid production in regulated secretory vesicles of neuronal chromaffin cells: evidence for cathepsin B as a candidate beta-secretase of Alzheimer’s disease. Biol Chem.

[19] Felbor U (2002). Neuronal loss and brain atrophy in mice lacking cathepsins B and L. Proc Natl Acad Sci USA.

[20] Mueller-Steiner S (2006). Antiamyloidogenic and neuroprotective functions of cathepsin B: implications for Alzheimer’s disease. Neuron.

[21] Moon HY (2016). Running-Induced Systemic Cathepsin B Secretion Is Associated with Memory Function. Cell Metab.

[22] Kuhn HG, Dickinson-Anson H, Gage FH (1996). Neurogenesis in the dentate gyrus of the adult rat: age-related decrease of neuronal progenitor proliferation. J Neurosci.

[23] Erickson KI (2010). Brain-derived neurotrophic factor is associated with age-related decline in hippocampal volume. J Neurosci.

[24] Spirduso WW, Clifford P (1978). Replication of age and physical activity effects on reaction and movement time. J Gerontol.

[25] Voss MW (2013). Neurobiological markers of exercise-related brain plasticity in older adults. Brain Behav Immun.

[26] van Uffelen JG, Chin APMJ, Hopman-Rock M, van Mechelen W (2008). The effects of exercise on cognition in older adults with and without cognitive decline: a systematic review. Clin J Sport Med.

[27] Nagamatsu LS, Handy TC, Hsu CL, Voss M, Liu-Ambrose T (2012). Resistance training promotes cognitive and functional brain plasticity in seniors with probable mild cognitive impairment. Arch Intern Med.

[28] Baker LD (2010). Aerobic exercise improves cognition for older adults with glucose intolerance, a risk factor for Alzheimer’s disease. J Alzheimers Dis.

[29] Norton S, Matthews FE, Barnes DE, Yaffe K, Brayne C (2014). Potential for primary prevention of Alzheimer’s disease: an analysis of population-based data. Lancet Neurol.

[30] Ingles, M. *et al*. Active paraplegics are protected against exercise-induced oxidative damage through the induction of antioxidant enzymes. *Spinal Cord* (2016). 10.1038/sc.2016.5 26882488

[31] Lezak, M. D., Howieson, D. B. & Lorin™g, D. W. *Neuropsychological assessment*. 4th ed./Lezak, M. D., Howieson, D. B., Loring, D. W. with Hannay, H. J. and Fischer, J. S. edn (Oxford University Press, 2004).

[32] Wechsler D (1945). A standardized memory scale for clinical use. The Journal of Psychology.

[33] Grober E, Buschke H, Crystal H, Bang S, Dresner R (1988). Screening for dementia by memory testing. Neurology.

[34] Pena-Casanova J (2009). Spanish Multicenter Normative Studies (NEURONORMA Project): norms for the Rey-Osterrieth complex figure (copy and memory), and free and cued selective reminding test. Arch Clin Neuropsychol.

[35] Tulving E, Osler S (1968). Effectiveness of retrieval cues in memory for words. J Exp Psychol.

[36] Petersen RC, Smith GE, Ivnik RJ, Kokmen E, Tangalos EG (1994). Memory function in very early Alzheimer’s disease. Neurology.

[37] Grober E, Lipton RB, Hall C, Crystal H (2000). Memory impairment on free and cued selective reminding predicts dementia. Neurology.

[38] Chan KL, Tong KY, Yip SP (2008). Relationship of serum brain-derived neurotrophic factor (BDNF) and health-related lifestyle in healthy human subjects. Neurosci Lett.

[39] Ngandu T (2015). A 2 year multidomain intervention of diet, exercise, cognitive training, and vascular risk monitoring versus control to prevent cognitive decline in at-risk elderly people (FINGER): a randomised controlled trial. Lancet.

[40] Polidori, M. C., Nelles, G. & Pientka, L. Prevention of dementia: focus on lifestyle. *Int J Alzheimers Dis* **2010** (2010). 10.4061/2010/393579 PMC2915647 20721289

[41] Ferris LT, Williams JS, Shen CL (2007). The effect of acute exercise on serum brain-derived neurotrophic factor levels and cognitive function. Med Sci Sports Exerc.

[42] Ruscheweyh R (2011). Pain is associated with regional grey matter reduction in the general population. Pain.

[43] Grober E, Sanders AE, Hall C, Lipton RB (2010). Free and cued selective reminding identifies very mild dementia in primary care. Alzheimer Dis Assoc Disord.

[44] Stricks L, Pittman J, Jacobs DM, Sano M, Stern Y (1998). Normative data for a brief neuropsychological battery administered to English- and Spanish-speaking community-dwelling elders. J Int Neuropsychol Soc.

[45] Campo P, Morales M (2004). Normative data and reliability for a Spanish version of the verbal Selective Reminding Test. Arch Clin Neuropsychol.

[46] Grober E, Lipton RB, Katz M, Sliwinski M (1998). Demographic influences on free and cued selective reminding performance in older persons. J Clin Exp Neuropsychol.

[47] Cotman CW, Berchtold NC (2002). Exercise: a behavioral intervention to enhance brain health and plasticity. Trends Neurosci.

[48] Katoh-Semba R (2007). Age-related changes in BDNF protein levels in human serum: differences between autism cases and normal controls. Int J Dev Neurosci.

[49] Cho HC (2012). The concentrations of serum, plasma and platelet BDNF are all increased by treadmill VO(2)max performance in healthy college men. Neurosci Lett.

[50] Castellano V, White LJ (2008). Serum brain-derived neurotrophic factor response to aerobic exercise in multiple sclerosis. J Neurol Sci.

[51] Currie J, Ramsbottom R, Ludlow H, Nevill A, Gilder M (2009). Cardio-respiratory fitness, habitual physical activity and serum brain derived neurotrophic factor (BDNF) in men and women. Neurosci Lett.

[52] Gomez-Cabrera MC (2006). Oxidative stress in marathon runners: interest of antioxidant supplementation. Br J Nutr.

[53] Sanchis-Gomar F (2015). Allopurinol prevents cardiac and skeletal muscle damage in professional soccer players. Scand J Med Sci Sports.

[54] Ji LL, Gomez-Cabrera MC, Vina J (2006). Exercise and hormesis: activation of cellular antioxidant signaling pathway. Ann N Y Acad Sci.

[55] Clow C, Jasmin BJ (2010). Brain-derived neurotrophic factor regulates satellite cell differentiation and skeltal muscle regeneration. Mol Biol Cell.

[56] Loprinzi PD, Frith E (2019). A brief primer on the mediational role of BDNF in the exercise-memory link. Clin Physiol Funct Imaging.

[57] Kim, S. *et al*. Roles of myokines in exercise-induced improvement of neuropsychiatric function. *Pflugers Arch* (2019). 10.1007/s00424-019-02253-8 30627775

[58] Delezie J, Handschin C (2018). Endocrine Crosstalk Between Skeletal Muscle and the Brain. Front Neurol.

[59] Vina J, Lloret A, Orti R, Alonso D (2004). Molecular bases of the treatment of Alzheimer’s disease with antioxidants: prevention of oxidative stress. Mol Aspects Med.

[60] Vina J, Borras C, Gomez-Cabrera MC (2018). A free radical theory of frailty. Free Radic Biol Med.

[61] Cassilhas RC (2007). The impact of resistance exercise on the cognitive function of the elderly. Med Sci Sports Exerc.

[62] Colcombe SJ, Kramer AF, McAuley E, Erickson KI, Scalf P (2004). Neurocognitive aging and cardiovascular fitness: recent findings and future directions. J Mol Neurosci.

[63] Liu-Ambrose T, Nagamatsu LS, Voss MW, Khan KM, Handy TC (2012). Resistance training and functional plasticity of the aging brain: a 12-month randomized controlled trial. Neurobiol Aging.

[64] Maass A (2015). Relationships of peripheral IGF-1, VEGF and BDNF levels to exercise-related changes in memory, hippocampal perfusion and volumes in older adults. Neuroimage.

[65] Voelcker-Rehage C, Godde B, Staudinger UM (2011). Cardiovascular and coordination training differentially improve cognitive performance and neural processing in older adults. Front Hum Neurosci.

