# Supplementary material for: Long-term exercise training improves memory in middle-aged men and modulates peripheral levels of BDNF and Cathepsin B
Source: Sci Rep. 2019 Mar 4;9:3337. doi: 10.1038/s41598-019-40040-8 (PMC6399244; doi:10.1038/s41598-019-40040-8)
Supplement: Supplementary file 1 — Supplementary Figure 1 [file 41598_2019_40040_MOESM1_ESM.docx]

Title: Long-term exercise training improves memory in middle-aged men and modulates peripheral levels of BDNF and Cathepsin B.

Authors: Adrian De la Rosa^1†^, Elisabeth Solana^2†^, Rubén Corpas^3†^, David Bartres-Faz^2^, Mercè Pallàs^4^, JoseVina^1^, Coral Sanfeliu^3^*^#^, Mari Carmen Gomez-Cabrera^1^*^#^

A B


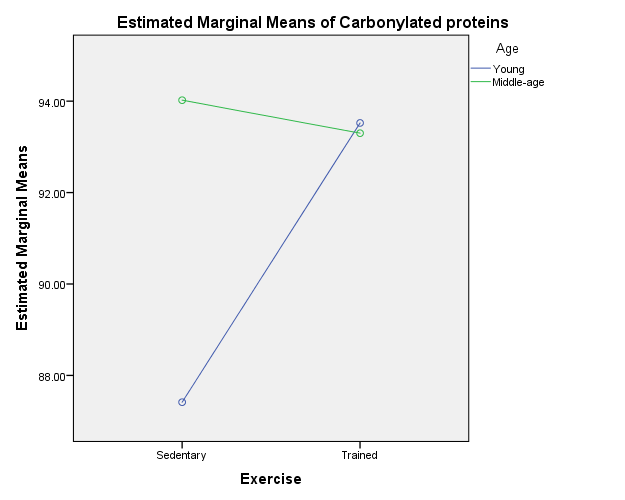

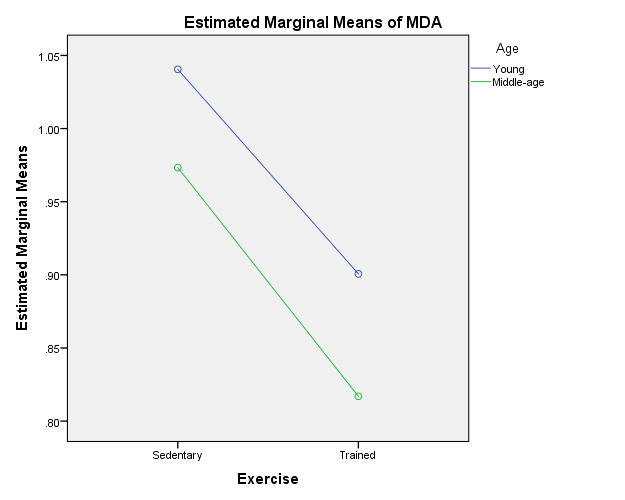


C D


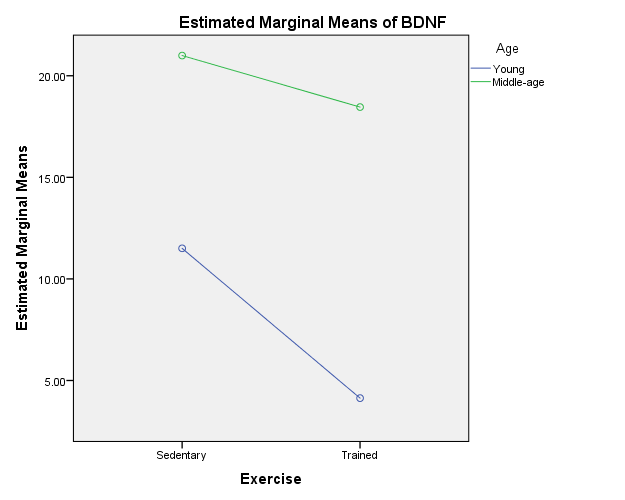

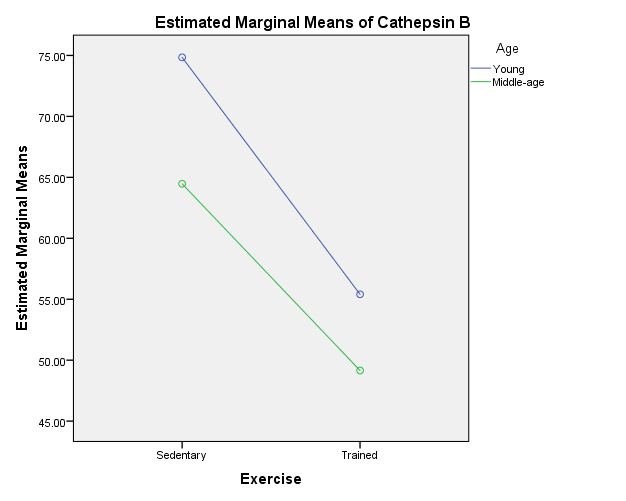


**Supplementary Figure 1.**

ANOVA plots displaying the presence of interaction between Age and Exercise factors for each variable of the study analyzed in peripheral blood samples. (A) Carbonylated proteins, non-statistically significant trend; (B) Malondialdehyde (MDA), parallel lines at both ages indicating lack of interaction; (C) Brain derived neurotrophic factor (BDNF), divergent lines showing interaction between Age and Exercise training; and (D) Cathepsin B, showing lack of significant interaction.
